# Exploring the Conformational Impact of Glycine Receptor TM1-2 Mutations Through Coarse-Grained Analysis and Atomistic Simulations

**DOI:** 10.3389/fmolb.2022.890851

**Published:** 2022-06-28

**Authors:** Anil Ranu Mhashal, Ozge Yoluk, Laura Orellana

**Affiliations:** Protein Dynamics and Cancer Lab, Department of Oncology-Pathology, Karolinska Institute, Solna, Sweden

**Keywords:** glycine receptor (GlyR), mutations, hyperekplexia, cancer, molecular dynamics, coarse-grained (CG) methods

## Abstract

Pentameric ligand-gated ion channels (PLGICs) are a family of proteins that convert chemical signals into ion fluxes through cellular membranes. Their structures are highly conserved across all kingdoms from bacteria to eukaryotes. Beyond their classical roles in neurotransmission and neurological disorders, PLGICs have been recently related to cell proliferation and cancer. Here, we focus on the best characterized eukaryotic channel, the glycine receptor (GlyR), to investigate its mutational patterns in genomic-wide tumor screens and compare them with mutations linked to hyperekplexia (HPX), a Mendelian neuromotor disease that disrupts glycinergic currents. Our analysis highlights that cancer mutations significantly accumulate across TM1 and TM2, partially overlapping with HPX changes. Based on 3D-clustering, conservation, and phenotypic data, we select three mutations near the pore, expected to impact GlyR conformation, for further study by molecular dynamics (MD). Using principal components from experimental GlyR ensembles as framework, we explore the motions involved in transitions from the human closed and desensitized structures and how they are perturbed by mutations. Our MD simulations show that WT GlyR spontaneously explores opening and re-sensitization transitions that are significantly impaired by mutations, resulting in receptors with altered permeability and desensitization properties in agreement with HPX functional data.

## Introduction

**FIGURE 1:**
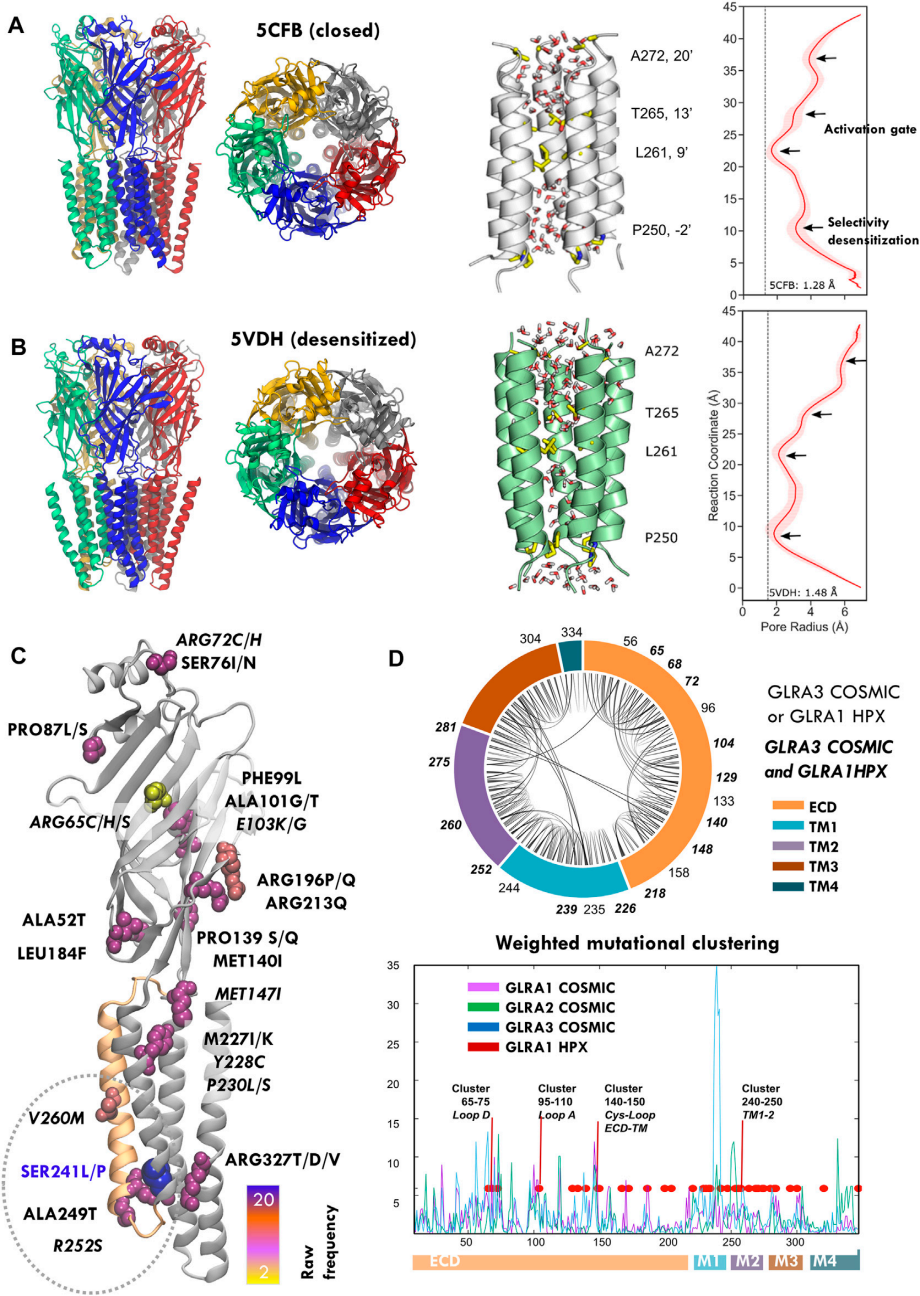
Structure and mutations of the glycine receptor (GlyR). **(A,B)** Human GlyR closed (5cfb) and desensitized (5vdh) structures used as reference in this study (*left*), zoom onto TM2 pore helices with key pore residues highlighted with licorice representation (*center*) and corresponding profile of pore radii calculated by HOLE (*right*). **(C)** COSMIC GLRA3 mutations colored by frequency; note the top mutation in blue (S241L) at the end of pore lining TM2 (orange). **(D)** Chord plot of the network of conserved contacts from 5CFB, with doubly HPX-COSMIC mutated residues in bold (top) and mutational clustering across GLRA1-3 genes weighted by conservation score (bottom). Note how multiple peaks overlap with positions mutated in startle disease (*red*) and the highest one corresponds to the TM1-2 region where HPX changes concentrate. See related Supplementary Figure S1 *,* Supplementary Table S1.

Pentameric ligand-gated ion channels (PLGICs) form a large family of integral membrane proteins with a central role in signal transduction from prokaryotes to eukaryotes (Dacosta and Baenziger, 2013; Changeux, 2014; Taly et al., 2014). Their ring-like pentamer architecture, with a fivefold symmetry axis centered on the ion-conducting pore, is conserved from bacteria to humans and mediates an incredibly sophisticated mechanism to allosterically propagate signals from the extracellular binding site to an ionic gate situated up to 50 Å away. In animals, PLGICs share a conserved extracellular cysteine bridge, which gives its name to the so-called Cys-loop family of ionotropic receptors. Given their key role in chemical synapses, Cys-loop receptors are major drug targets in neurological conditions from Alzheimer to rare genetic diseases. Mostly expressed at post-synaptic neurons, upon pre-synaptic neurotransmitter release, they mediate passive ion fluxes that shift the membrane potential. Depending on pore-lining residues, PLGICs are selective for cations like sodium ions (Na^+^) and result in excitatory effects, e.g. the nicotinic acetylcholine receptor (nAch-R) or the serotonin type-3 receptor (5-HT_3_-R), or are selective for anions like chloride ions (Cl^−^) resulting in inhibitory effects, e.g., the α-aminobutyric acid receptor (GABA_A_-Rs) or the glycine receptor (Gly-Rs) (Figure 1).

Whether prokaryotic or eukaryotic, PLGICs share a topology characterized by a large N-terminal ligand-binding extracellular domain (ECD), followed by four transmembrane domains (TM1–4). Monomers assemble into a pentameric cylinder, with the five orthosteric ligand binding sites (LBS) located at ECD subunit interfaces, TM2 helices shaping the ion channel pore across the central symmetry axis, and TM4 helices facing the plasma membrane (Figures 1A,B, left). This universal topology is linked to incredibly conserved functional mechanisms to regulate ion gating (Gielen and Corringer, 2018). Classically, PLGIC activation was interpreted with simple two-state models like the MWC (Monod et al., 1965), in which receptors spontaneously sample resting/shut and active/open states until agonist binding shifts the equilibrium. Nevertheless, single-channel studies and growing structural data show a far more complex conformational cycle, with striking similarities across species. In the initial pre-activation or “priming” step, agonist binding stabilizes the ECD in a contracted higher-affinity conformation (“*un-blooming*”), triggering a key revolving motion of TM2-3 loop at the ECD–TMD interface and subsequent ECD–TMD rotation in opposite directions (“quaternary twist”) (Nemecz et al., 2016). This sequence of events is captured by the so-called “locally closed” conformations, where the ECD has undergone the transition toward the active state-like conformation, but the TMD still remains in a resting conformation (Prevost et al., 2012). In the activation step, global twisting couples to cooperative tilting of pore-lining helices (“iris-like gating”) (Martin et al., 2017), which widens the upper part of the channel (“activation gate”, TM2 9′ and 13′; see Figures 1A,B right), formed by two or three rings of hydrophobic residues that create a barrier to ion permeation (Althoff et al., 2014; Du et al., 2015). Once the gate is open, ions flow according to their electrochemical gradient and channel selectivity, determined by the “selectivity filter” at the cytoplasmic end of the pore (TM2 -1′ or -2′). Remarkably, this “unbloom-and-twist” allosteric mechanism that propagates a signal (ligand) from the ECD to a remote TMD pore gate is encoded in the PLGIC fold, as predicted by elastic network models (ENMs) (Bahar et al., 2010), and it has been further confirmed by molecular dynamics (MD) (Calimet et al., 2013) and coarse-grained eBDIMS simulations (Orellana et al., 2016). Apart from agonist-elicited activation, PLGICs also share another key physiological property: desensitization, in which the sustained agonist presence causes the channels to transit from the active to an agonist-bound inactive state (Katz and Thesleff, 1957) to prevent over-activation. Once the agonist disappears, receptors slowly recover, although the detailed transitions from agonist-unbound desensitized states to unbound resting or open states are still unclear. Desensitization mainly involves pore closure at its intracellular end (TM1-2 loop), which therefore also acts as the main “desensitization gate” (Hibbs and Gouaux, 2011).

Among Cys-loop receptors, strychnine-sensitive GlyR, the major inhibitory ionotropic receptor in the brainstem and spinal cord, has become by far the better characterized (Howard, 2021): there are over 40 structures deposited in the Protein Data Bank, mostly in open, closed and desensitized-like states (Du et al., 2015; Huang et al., 2015, 2017b, 2017a; Kumar et al., 2020; Yu J. et al., 2021, Yu et al., 2021 H.); a few are trapped in a super-open states, whose physiological significance has been questioned, specially by MD (Cerdan et al., 2018; Cerdan and Cecchini, 2020; Dämgen and Biggin, 2020; Dämgen et al., 2020). Apart from these rich structural data, GlyR stands out among the eukaryotic PLGICs due to its role in neurological diseases and particularly in a rare Mendelian condition known as hyperekplexia (HPX) or “startle disease” (Lynch et al., 2017). Similar to the way the alkaloid strychnine antagonizes glycine binding, hyperekplexia disrupts glycinergic neurotransmission, resulting in exaggerated “startle” responses and muscle stiffness. Analysis of hyperekplexia patients has resulted in an exceptional amount of information on GlyR mutations and their phenotypic impact being gathered during the past two decades (Lewis et al., 1998; Chung et al., 2010; Bode et al., 2013; Bode and Lynch, 2014). As the GABA receptor, GlyR usually functions as an heteropentamer of alpha and beta subunits, but only alpha subunits form functional homopentamers. Over 50 mutations have been linked to hyperekplexia, mostly targeting the alpha1 subunit (GLRA1), thus providing an exhaustive mutational scanning map in terms of structural areas where missense changes result in GlyR disruption (Chung et al., 2010; Bode et al., 2013; Bode and Lynch, 2013, 2014). In general, HPX mutations (Supplementary Table S1, Figure 1D) are either recessive and typically associated with low surface expression (loss-of-function), or dominant, mostly located around TM2 and causing prolonged desensitization and/or spontaneous activation (gain-of-function), which leads to reduced maximal currents.

Despite the fact that its central function is synaptic signaling, PLGICS are also expressed in non-neural cells where they play a diversity of roles, including stem cell and cancer proliferation (Young and Bordey, 2009; Zhang et al., 2013; Bhattacharya et al., 2021). GlyR is no exception and is known to be expressed in cells as diverse as hepatocytes, spermatozoa, pancreatic, endothelial, or renal cells (Lynch, 2004; Van den Eynden et al., 2009). As tumor genomic screenings advance, an increasing number of GlyR mutations are being reported in a surprising variety of tumors. For the three GlyR genes, GLRA1-3, cancer-reported mutations display an intriguing clustering partially overlapping with HPX positions (Figures 1C,D, Supplementary Table S1). Here, we perform a preliminary exploration of selected GlyR mutations using as model the GLRA3 homomer, which apart of being structurally well characterized in humans also carries the highest frequency of mutations, specially focused on the lower TM2 section (Figure 1C). Based on mutational clustering, conservation, and ENM analysis, we select for MD study three TM1 and 2 mutations found in tumors, which are close or overlap with hyperekplexia-mutated positions (S241L, R252S, and V260M) of uncertain functional effect. Our results suggest that these residue substitutions are far from neutral but profoundly impair channel permeability, selectivity, and desensitization.

## Methods

### Structural Data, Sequence Alignment, and Conserved Network Analysis

The reference wildtype structures for closed (PDB ID: 5CFB, Figure 1A) and desensitized state (PDB ID: 5VDH; Figure 1B) human GlyR GLRA3 homopentamer were obtained from the Protein Data Bank and the series of mutants, S241L, R252S, and V260M were constructed from both states, resulting in eight systems. To select candidate mutations for further study, we focused on the resting 5CFB structure to perform a simple weighting analysis based on the 3D space distribution and degree of conservation as in Orellana et al. (2019b). First, we fetched the information for GLRA1-3 missense mutations reported in the COSMIC database (Tate et al., 2019) (Supplementary Figures S1, S2). Conservation scores were retrieved from the ConSurf database (Ashkenazy et al., 2016); to get a simpler overview of residue conservation, sequences for GLRA1-3, GBRA1, and the intensely studied PLGICS *G.violaceus* GLIC and *C. elegans* GluCL were retrieved from the UniProt database (The UniProt Consortium, 2021) and aligned with ClustalW (Supplementary Figure S1). Then, in order to evaluate spatial 3D clustering, we applied a simple counting algorithm: each amino acid is represented by its C-alpha carbon, and the number of mutations reported for each position and its neighbors within a 3D-sphere of cutoff radius 9 Å (typical to evaluate residue pairwise interactions) is added to obtain a raw number of hits (i.e., reported mutations within the 3D-sphere) (Supplementary Figure S2A), which are then weighted according to their *ConSurf* scores (Figure 1D, bottom). Hence, random isolated mutations or changes in non-conserved areas are filtered out to obtain a final estimate of the conserved spatial mutation concentration around each amino acid. Finally, the contact network between highly conserved residues (i.e., those with *ConSurf* scores 8–9) was plotted with a chord diagram to visualize interactions across different regions and mutated areas (Figure 1D, top).

### Principal Component Analysis

**FIGURE 2:**
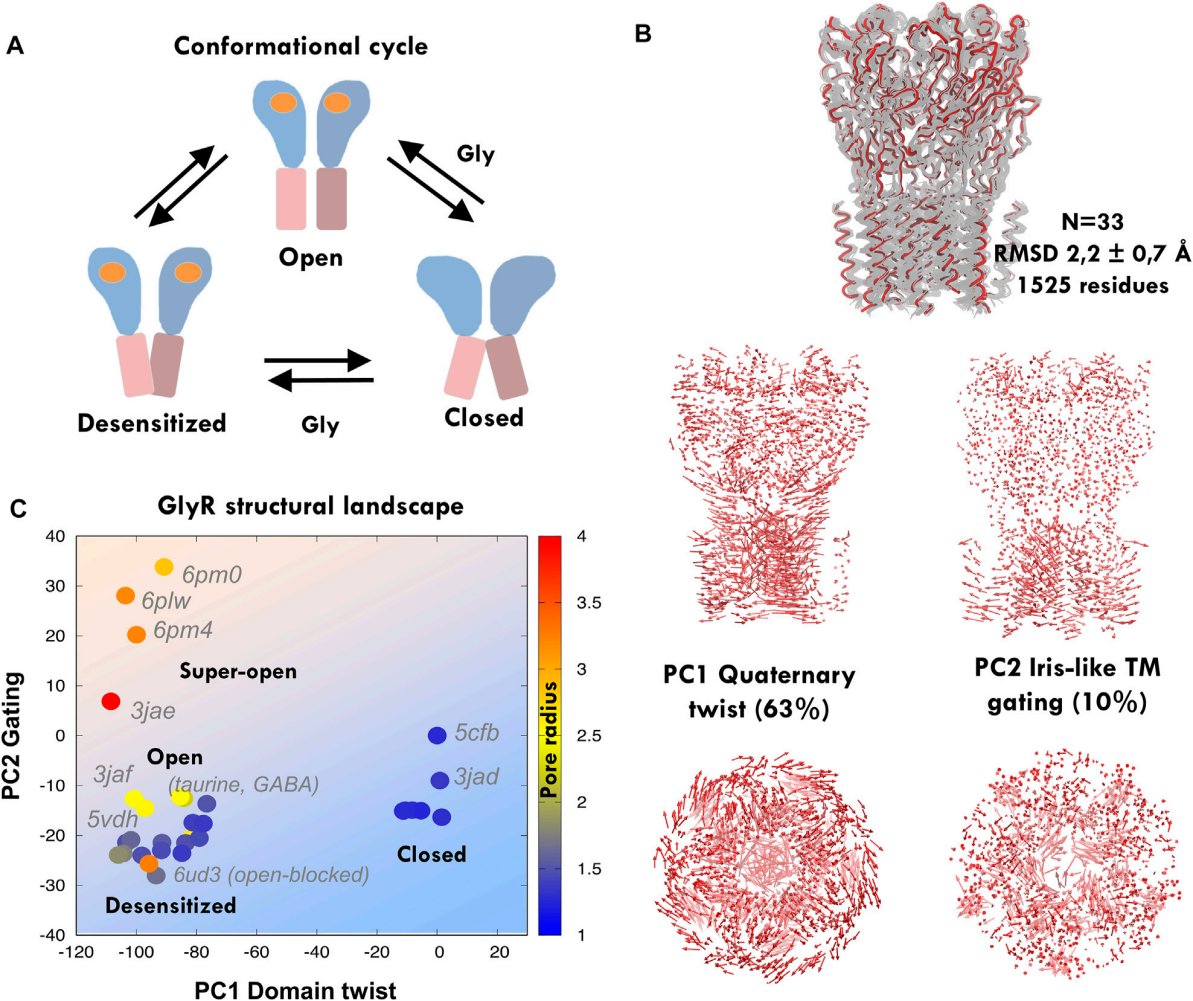
Principal component analysis and elastic network modeling of GlyR. **(A)** Simplified scheme of the conformational cycle depicting the three main meta-stable states: closed, open, and desensitized, with ECD in blue and TMD in pink depicting blooming and open and closed pore TM2 orientations (see the main text). **(B)** Experimental GlyR ensemble containing human and zebrafish structures (*top*) and principal components from the GlyR ensemble (*bottom*): the major component tracks the anticorrelated ECD-TMD twist, while the second captures iris-like pore gating. **(C)** Projections of the GlyR ensemble onto PC1-2, with structures colored according to their radius. Note how structures separate onto three clusters corresponding to the assigned functional states, with the only exception of open blocked 6ud3. The human closed structure *5cfb* is used as a reference and thus projects at the origin. See related Supplementary Figure S1 *,* Supplementary Table S1 *.*

Principal component analysis (PCA) (Jolliffe, 2002) is a statistical technique to reveal dominant patterns in noisy data. The diagonalization of the covariance matrix of the system allows obtaining the major axis for statistical variance or principal components (PCs). In this way, complex multidimensional data are mapped to a reduced set of coordinates, which contain the dominant trends explaining data variation. PCA has been widely applied in structural biology to analyze ensembles, usually coming from MD simulations (Amadei et al., 1993, 1996). Protein structures are aligned to a reference in order to compute a covariance matrix, which describes the mean-square deviations in atomic coordinates from their mean position (diagonal elements) and the correlations between their pairwise fluctuations (off-diagonal elements). Diagonalization then yields a set of eigenvectors (principal components, PCs) and eigenvalues representing the motions that explain the variation in the atomic coordinates. In the structurally rich ensembles here analyzed, the first two PCs contain, on average, around 60–80% of the ensemble structural variation (Orellana et al., 2016), and provide excellent coordinates to assess mutation effects on MD sampling (Chen et al., 2021a; 2021b). On this framework, a structure *i* containing N residues is thus accurately characterized by its projections onto the conformational space defined by the major components, PC_k_ (k = 1, 2 .… 3N-6) (see Figure 2)

$$PC_{k}=\;\left[T_{i-0}\right]\cos\alpha \;.$$ (1)

where T _i-0_ is the difference between the coordinates of *i*-structure and *the apo reference,* PC is one of the major axes, and α is the angle formed by PC_k_ and T _i-0_. Here, we retrieve all available GlyR structures in the Protein Data Bank, corresponding to *H. sapiens, D. rerio,* and *S. domesticus*, and after elimination of structures with missing gaps and alignment to the common conserved core, we obtain an ensemble of 33 structures (*3jad, 3jae, 3jaf, 5cfb, 5tin, 5vdh, 5vdi, 6plo. 6plp, 6plq, 6plr, 6pls, 6plt, 6plu, 6plv, 6plw, 6plx, 6ply, 6plz, 6pm0, 6pm2-6, 6ubs, 6ubt, 6ud3, 6vm0, 6vm2-3, 7mlu*) that aligned to 5cfb with low RMSD. As previously shown by us (Orellana et al., 2016), major PCs are captured, i.e., correlate with the heuristic variables that typically characterize PLGIC conformations (Supplementary Figure S2B). Projections were used to also track the time evolution of trajectories (Figure 4).

### Molecular Dynamics Simulations and Essential Dynamics

**FIGURE 3:**
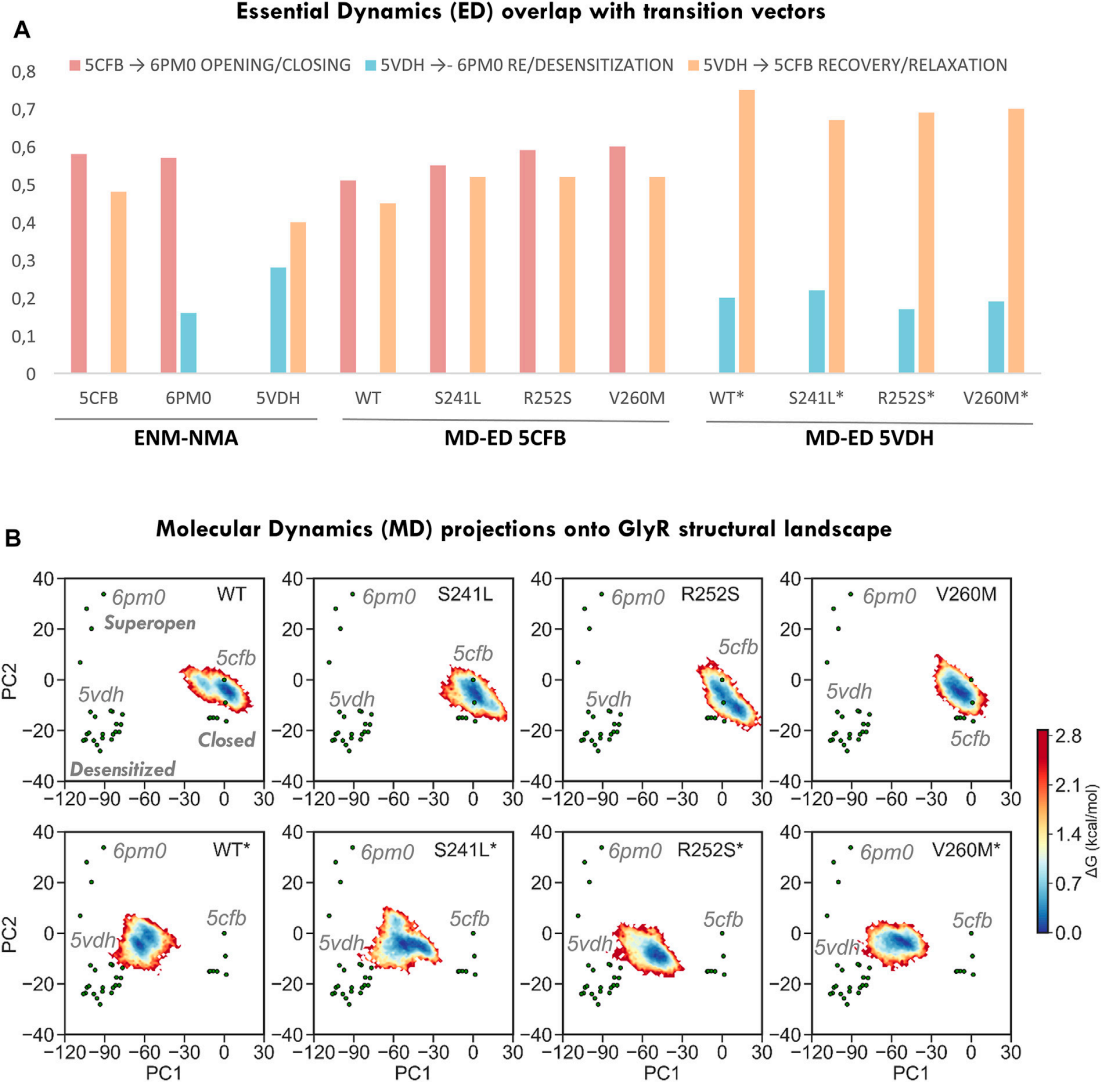
Conformational space sampling by elastic network models and essential dynamics across the three major axis for transition defined by closed (5CFB), desensitized (5VDH), and super-open (6PM0) structures from each PC cluster. **(A)** Overlap between the first 10 ENM modes computed from the three state representatives versus the three transitions (left) and between the first 10 ED modes from 5CFB and 5VDH simulations versus the three transitions (right). **(B)** Relative free energy landscapes for 1.2 us meta-trajectories upon projection onto PC1-2. Note how simulations from 5CFB (top row) extend in the direction of super-open structures, and while for WT GlyR populate two minima, for mutations sample an elongated one. On the contrary, simulations from 5VDH evolve toward the closed cluster, while for WT GlyR they point to 5cfb, mutants tend to evolve to collapsed closed states that project at negative PC2 values. All simulations from 5VDH are indicated with *.

We used the CHARMM-GUI web interface to build systems for MD simulation (Jo et al., 2008, 2017), which allowed us to repair missing residues in the crystal structures and build a membrane bilayer containing ∼300 phospholipid POPC (1-palmitoyl-2-oleoyl-sn-glycero-3-phosphocholine) molecules. A hexagonal water box was used to reduce the cell volume and overall system size. Membrane-embedded proteins were then solvated using the TIP3P water model. The CHARMM36m force field was used to describe the system (Huang et al., 2016). Potassium (K^+^) and chloride (Cl^−^) ions were added to maintain the physiological salt (150 mM KCl) concentration to mimic intracellular conditions. Energy minimization, equilibration, and production runs were carried out with GROMACS (Pronk et al., 2013; Abraham et al., 2015), following the CHARMM-GUI Membrane Builder standard protocols (Wu et al., 2014). The temperature was maintained at 303.0 K using the Nose–Hoover thermostat (Nosé, 1984; Hoover, 1985) and pressure was set to 1.0 bar using the Parrinello–Rahman barostat (Parrinello and Rahman, 1981) with semi-isotropic pressure coupling. Hydrogen bonds were constrained using the LINCS algorithm (Hess et al., 1997), short-range van der Waals (vdW), and electrostatic interactions cutoffs were set to 12 Å, and long-range electrostatic interactions were described using the particle mesh Ewald approach (Ewald, 1921; Essmann et al., 1995) with periodic boundary conditions. Production runs were carried out using a 2 fs time step and writing at every 1 ps interval. Each system was simulated for 300 ns, with four replicas starting from different random seeds. Therefore, in total, we simulated 1.2 μs for each one of the eight GlyR systems, i.e., from 5CFB/5VDH, with WT/S241L/R252S/V260M sequences. To filter out noise and extract the main collective motions, we performed *essential dynamics* (ED) (Amadei et al., 1993; Daidone and Amadei, 2012) for each system’s 1.2 μs meta-trajectory (Figure 3A) using in-house scripts. The relative free energy landscape (FEL) at 300 K was obtained from the probability distribution of the reaction coordinate, R (PC1, PC2) (Figure 3B). GROMACS tools with defaults were used to perform the RMSD cluster analysis of the TMD and calculate the average TM1-2 helicity. For cluster analysis (Figure 8), all simulated systems were combined at a 1 ns interval to yield a single and long C_α_ atom 9.6 μs trajectory of 9600 frames, and an RMSD cut-off of 0.20 nm was selected to obtain lesser and larger clusters. See the summary of all simulations in Supplementary Table S2.

### Elastic Network Normal Mode Analysis

Elastic network models (ENM) (Atilgan, 2018) are minimal coarse-grained representations of protein structures as beads-and-springs. In the anisotropic network model (ANM), the potential energy of a protein structure—assumed to be at an energy minimum—is defined by a network of interactions with nodes at the C^α^ atoms coupled by uniform springs (Tirion, 1996; Atilgan et al., 2001). Once the pair force constants *K*_*ij*_ are defined, the network potential energy is approached by the parabola:

$$E=\;\sum_{i=j}K_{\text{ij}}{\left(R_{\text{ij}}-R_{ij}^{0}\right)}^{2}$$ (2)

where R_ij_ and R_ij_^0^ are the instantaneous and equilibrium distances between all nodes. Within normal mode analysis (Case, 1999), the Hessian matrix of the energy second derivatives is diagonalized, yielding a set of orthonormal 3N-6 eigenvectors representing the normal modes, which have been shown to accurately predict intrinsic collective motions. Here, we use the nearest-neighbors MD-derived ED–ENM algorithm, which predicts experimental conformational changes (Orellana et al., 2010) and, implemented onto ED–ENM Brownian dynamics (eBDIMS), entire sequences of intermediate states along transition pathways (Orellana et al., 2016; 2019a). Average thermal fluctuations for each residue pair *i, j* and each residue *i* are evaluated as in Atilgan et al. (2001) (Supplementary Figure S2C).

### Essential Dynamics and Normal Mode Analysis Similarity Against Experimental Transition Vectors

An experimental conformational change between two conformations *R*_*1*_ and *R*_*2*_ can be described by the normalized difference 3N-dimensional vector *∆r =* (*R*_*2*_ *- R*_*1*_)/║*R*_*2*_ *- R*_*1*_║ between the two sets of alpha-carbon coordinates, after optimal superimposition of the structures. Therefore, given a motion space from ED or NMA, the degree of similarity or overlap between the directions of the experimental *∆r* vector and a given *k*th mode υ_k_ is measured by their angle cosinus (Yang et al., 2009):

$$\alpha_{k}=\;\frac{\Delta r.v_{k}}{\left\|\Delta r\right\|\left\|v_{k}\right\|}$$ (3)

where ∙ designates the dot product and the bars denote the vectors’ modulus or magnitude; a cosinus close to 1 means that the directions are parallel (Figure 3A). Therefore, the similarity between ANM or ED modes and the transition is evaluated by the cumulative contribution of the first M modes

$$\delta_{k}=\;{\left(\sum_{1}^{M}\alpha_{k}^{2}\right)}^{1/2}$$ (4)

Here, we consider M = 10, which typically cover >90% of the variance (Orellana et al., 2010) (Figure 2A).

### Heuristic Channel-Defined Structural Variables

To evaluate channel descriptors as in Orellana et al. (2016) (Figures 5–7, Supplementary Table S3, S4), we used VMD along with in-house tools and scripts written in *python*, C++, and FORTRAN. The *quaternary twist* motion is the anti-correlated rotational movement of the ECD versus TMD around the channel axis, which decreases as the channel transitions to the open state. Channel closing is also coupled to ECD collapse (un-bloom) to a narrower diameter. The iris-like gating motion can be broken down into two components in the form of tilt and twist motions of the M2 helices that alter the pore radius. Hence, *blooming* was evaluated as the maximal radius of the extracellular domain defined by maximally separated residues at the tip of the five subunits. *Quaternary twist* was calculated as the average rotation angle of each subunit with the vector from extracellular domain and transmembrane domain CM to overall CM on the XY plane. The *tilt and the twist angle of M2 helices* were calculated as an average over five subunits. In order to get comparable angles between subunits, the reference structure (5CFB) was aligned to the center of mass of each M2 helix. *X*-axis was characterized between two centers of mass: M2 helix and the protein center of mass. The *Z*-axis was chosen as the principal of inertia parallel to the symmetry axis. Finally, the *Y*-axis was defined as the vector normal to the XZ plane. With these axes, the tilt angle is calculated between the projected helical axis onto the XZ plane and the *Z*-axis and twist angle between the projected helical axis onto the YZ plane and *Z*-axis. Pore calculations were performed with HOLE (Smart et al., 1996) using 0.5 Å step from C-alpha atoms only. The pore radius at 9′ was averaged over a 2.5 Å window in both directions from the center of mass of 9’ residues. The average hole profiles were obtained for the structures during the production run using only M2 helices and are reported with their standard deviation. Hydration in the GlyR channel was quantified by calculating the number of water molecules inside the channel pore along the axis normal to the bilayer. To study the conductivity of the GlyR channel in wild type and mutant proteins, we identified the water/ions in the pore and their *z* axis coordinates (bilayer normal) at every snapshot and plotted them versus simulation time.

## Results and Discussion

### GlyR Cancer Mutations and Dominant Hyperekplexia Changes Partially Overlap on TM1-2

As of March 2022, there are around 1000 cases of tumors reported to carry GlyR mutations: 952 for GLRA1, 1196 for GLRA2, and up to 1546 for GLRA3. After removing nonsense and deletion changes, we retrieved 245, 255, and 299 mutations, for GLRA1-3, respectively. Mutations for GLRA1 concentrate in 175 positions (70%), for GLRA2 in 174 (68%), and for GLRA3 in 208 (69%), which represent approximately 50–60% of the GlyR chain (347 positions); a majority of these changes are considered to be passenger mutations due to defective repair mechanisms in neoplastic cells. Nevertheless, as the mutations are not evenly distributed along GLRA sequences but preferentially focused on key conserved regions (Figures 1C,D, Supplementary Figure S1, Supplementary Table S1), we hypothesized they could impact on the receptor function. Particularly, for GLRA3, mutation S241L has high frequency, with over 20 cases reported, in contrast with top GLRA1 and GLRA2 mutations. Moreover, nearly half of the cases (12) of S241L mutation are linked to melanoma, followed by skin carcinoma (8), while in GLRA1-2, they tend to spread across multiple tumor types. A closer look also reveals specific changes recurrently appearing across GLRA1-3 genes and/or often overlapping with HPX mutations sometimes identical (see Figures 1C,D, Supplementary Table S1). Overall, these mutational patterns strongly suggest that at least a fraction of these changes could affect the channel function, which we decided to investigate in more detail for GLRA3, due to the availability of solved human X-ray structures and its significant mutational frequency. We focused our analysis on the resting state, represented by structure 5CFB. To filter out as many random changes as possible, we quantified mutation clustering (Figure 1D bottom, Supplementary Figure S2A) based on the 3D space distribution and degree of conservation as in Orellana et al. (2019b); we also mapped GLRA1-2 and hyperekplexia mutations to better detect overlapping patterns. We considered not only the raw number of mutations reported but also the recurrence of multiple allele changes onto the same positions (Supplementary Figure S2A). Upon weighting the spatial concentration of mutations by their *ConSurf* scores, we were able to identify four broad mutational clusters, which display overlap between hyperekplexia and cancer-reported GLRA1-3 changes: three minor ones at the ECD, located around loop D (residues 65–75), loop A (residues 95–110), and the Cys-loop ECD–TMD interface (residues 140–150), and a major one across TM1-2 (residues 222–244) to TM2 (residues 250–272); note that almost all reported HPX mutations overlap with one of these peaks with a few exceptions (Figure 1D bottom), with the notable exception of the most frequent mutation, R271L/Q/P, located at TM2-3 loop. Highest clustering scores were achieved at the area surrounding the TM1-2 loop region, where the selectivity filter and desensitization gate locate, and which contains HPX mutations such as P250T known to prolong desensitization (Saul et al., 1999). This area contains the highest frequency mutation, S241L/P (ConSurf score 8), located at the end of TM1, and its contacting neighbor R252S (ConSurf score 9), at the end of TM1-2 loop/beginning of TM2. While S241 is close to dominant hyperekplexia change W239C, of unknown functional effect, R252 is mutated in HPX as R252H/C, which results in low expression and activity. Another high-scoring neighbor area upstream TM2, close to 9′ gate, contains mutation V260M (ConSurf score 8), which corresponds exactly with a dominant HPX change (del Giudice et al., 2001; Castaldo et al., 2004) known to disrupt gating. Importantly, both R252 and V260 are located in the pore-opposing TM2 face, at the level of lower 2′ and upper 9′ gates (see Figures 1A,B, right). To further evaluate the significance of mutations, we also compared mutational patterns with the conserved contact networks of 5CFB (Figure 1D, top) and 5VDH (not shown). In contrast with S241 and R252, mostly involved in the local TM1-2 loop connectivity, V260 participates in longer-range intrachain contacts connecting TM2 with TM3; moreover, it also contacts TM2 (T262) across adjacent subunits in the closed but not the desensitized state (*not shown*). Overall, the concentration of these dramatic size, charge, and polarity changes overlapping HPX mutants near the conserved activation and desensitization gates suggests that they are not neutral but can impact receptor stability and conformation.

### GlyR Ensemble Encodes Opening and Desensitization Transitions

In spite of the diversified roles of PLGICs, structural studies have revealed an astonishing degree of fold and conformational conservation across species. Our previous studies of the channel GLIC and other model proteins show that the PCA of structural ensembles containing multiple conformations can reveal the pathways for inter-connecting transitions providing an accurate framework to monitor MD sampling (Orellana et al., 2016; Orellana, 2019). Currently, there are dozens of zebrafish and human GlyR structures solved in closed, desensitized, and open and super-open states (Figure 2A). We aligned *n* = 33 nearly-intact homo- and heteromeric structures (Figure 2B; RMSD = 2.2 ± 0.7 Å) and performed PCA to extract the dominant ensemble motions and then investigate how they correlate with channel descriptors and annotated functional status. Similar to what is observed for prokaryotic GLIC (Orellana et al., 2016), the first mode (PC1, 63% of the variance) tracks the global quaternary twist and blooming motion (R = 0.9 and 0.6), as well as TM2 tilt (R = −0.8), while the second mode (PC2, 10% of variance) captures most of TM2 twist and pore gating (R = 0.7 and 0.6) (Figure 2B, Supplementary Figure S2B), separating super-open from desensitized structures (see Methods for definitions). Together, PC1-2 (73% variance) split the structural ensemble onto three to four major clusters (Figure 2C): to the right, closed/antagonist-bound structures (pore radius ≈1.5 Å, 1.2 Å for 5CFB), and to the left, those with an un-bloomed ECD and a wide-open (top left corner, pore radius > 3 Å e.g., 6PMO) or desensitized central channel (lower left corner, ≈ 1.5 Å, 1.4 Å for 5VDH); the only exception is the open-blocked structure 6UD3. Significantly, structures annotated as open states (6PLO, 6PM2, 6PLY, etc.) appear correctly located as a sub-cluster along the path from fully desensitized to super-open structures (pore radius ≈2.5 Å), which supports that PC1-2 space is a suitable framework to annotate channel status based on global correlated features of multiple descriptors. Finally, we also investigated to which extent these large-scale gating movements coupled to opening/desensitization are intrinsic to the different GlyR states, as previously suggested (Bertaccini et al., 2010; Zhu and Hummer, 2010; Zheng and Auerbach, 2011). We took as representatives of the three main PC1-2 clusters our reference human closed (5CFB) and desensitized (5VDH), together with one of the super-open zebrafish structures (6PMO); these three structures altogether broadly define three main transition directions along which the conformational cycle could potentially proceed. ENM from these three distinct states (Figure 3A) indicates that while the opening transition could be fairly spontaneous in the absence of a ligand (overlap ≈60%), the same was less likely for re/desensitization. In contrast, normal modes computed from 5VDH displayed poor overlap with the associated transitions, suggesting a rigid structure with more uncorrelated local motions. Residue fluctuations from 5CFB–ENM (Supplementary Figure S2C) also indicated that these mutations are located in a transition region from high [TM1-2 loop (S241L)] to low flexibility (TM2, from R252S to V260M) and thus could have diverse effects despite their proximity, which we then explored with fully atomistic MD.

### Molecular Dynamics Reveals Mutations Perturb GlyR Conformational Dynamics

We performed MD simulations from the closed inhibited (PDB: 5CFB) and desensitized (PDB: 5VDH) human GLRA3 homopentamers after removing all ligands (the antagonist strychnine, in the first, and agonists glycine, AM-3607 and ivermectin in the latter), for WT and mutant sequences. Root-mean-square fluctuations (RMSDs) of the C_α_ atoms versus the initial experimental structures were calculated for all systems to confirm simulation convergence (Supplementary Figure S3A) and estimate the overall stability and flexibility of unbound closed and desensitized states upon ligand removal (Supplementary Figure S3B). Despite the fact that differences across replicates and GlyR variants were small, a closer look reveals a slightly more rigid desensitized state, with distributions shifted to lower values (Supplementary Figure S3B). This is often observed for bound/unbound systems and in agreement with our ENM preliminary analysis. Nevertheless, for both closed and desensitized state simulations, TM1-2 mutations in general shifted RMSD distributions to the right, suggesting increased conformational flexibility. While for R252S, the peak height is clearly shifted toward higher RMSDs suggesting greater thermal fluctuations, in V260M, the distribution spreads over multiple peaks suggestive of different conformational clusters. Interestingly, S241L displays virtually no difference versus WT GlyR for 5CFB simulations, in contrast with 5VDH simulations. Locally, the introduced mutations disrupt highly stable hydrogen bond and salt bridge WT interactions in both the closed and the desensitized receptors, both at the local TM1-2 level and globally (Supplementary Figures S4A,B). Significantly, a WT salt bridge between Asp247 and Arg252 present in 5CFB but absent in 5VDH is broken by all three mutations in the closed state. Among mutants, R252S has a major impact on the closed state introducing multiple non-native salt bridges not seen in any WT simulations far away from the mutation site, around TM3-4. Native contacts in this area, which are maintained in both 5CFB and 5VDH simulations, are the most perturbed by mutations; interestingly, V260M closed state simulations display interactions seen in the open-like/desensitized state like Glu300-Lys320. Similar changes are seen in hydrogen bond patterns, with mutations mostly affecting longer-range interactions with TM3 and TM4. Despite these profound reshaping of interactions, TM1-2 local helicity is mostly unaffected (Supplementary Figure S4C), only displaying transient and very limited unfolding in desensitized state simulations, focused at helix termini surrounding the TM1-2 loop junction.

**FIGURE 4:**
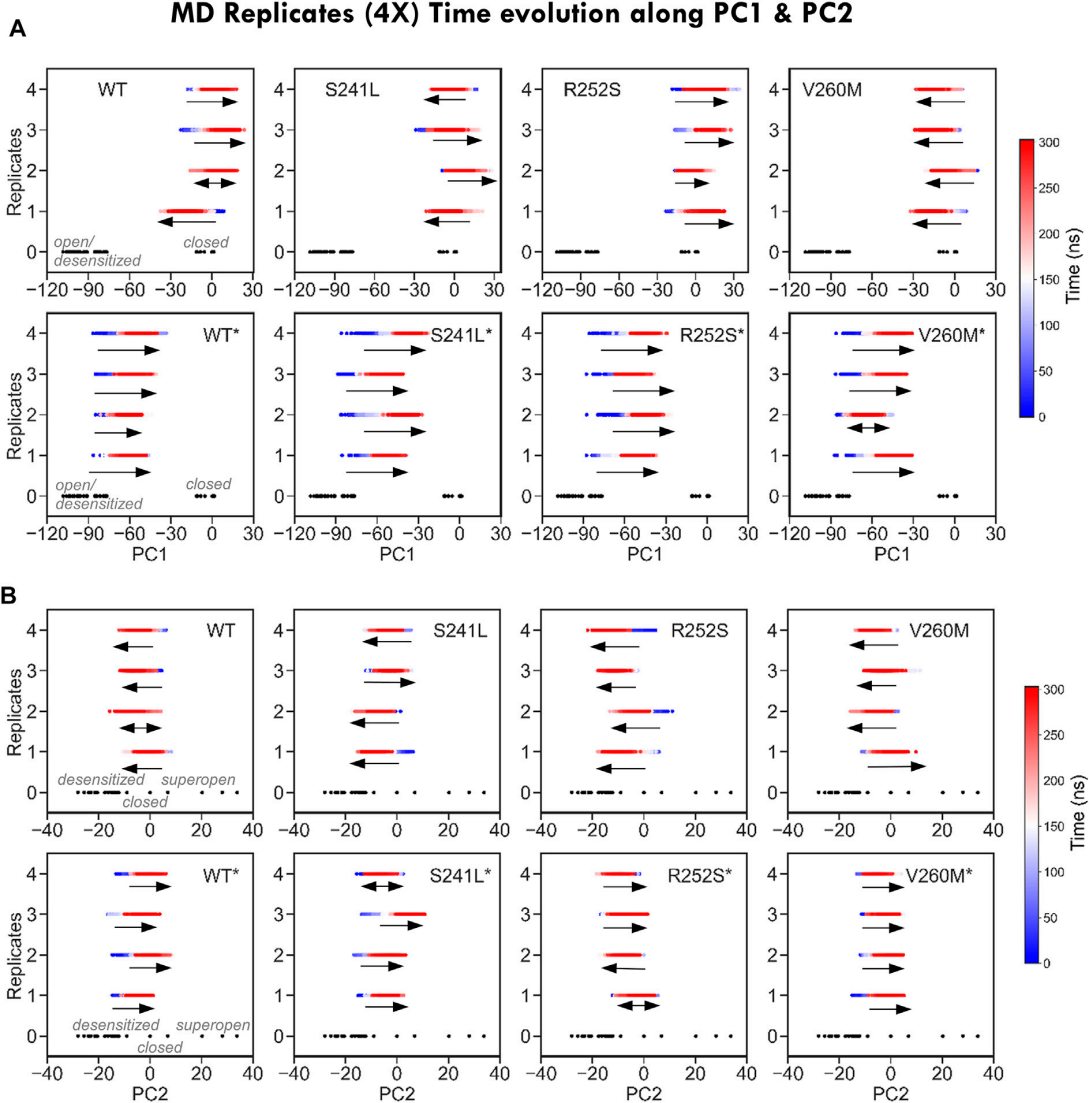
Time evolution of MD replicates along PC1 and PC2. **(A)** Time evolution of MD trajectories along PC1, which separates closed from open/desensitized structures, for each of the four replicates from each system. Note how for simulations from the closed state (*top*), trajectories evolve in both closed and open/desensitized directions or stay around the starting structure, with the exception of V260M, in which all runs proceed toward the open region to the left. On the contrary, all desensitized state simulations (*bottom*) progress in the closing direction. **(B)** Time evolution of MD trajectories along PC2, which separates closed from open/desensitized structures, for each of the four replicates from each system. Trajectories stay or evolve toward pore closing, with the exception of S241L and V260M, which have replicates progressing toward opening. On contrast, most simulations from the desensitized state (*bottom*) evolve toward pore opening, except for R252S*.

Given the long-range impact of mutations in H-bonding and salt bridge connectivity, we then explored their effect on global dynamics by extracting the MD *essential modes* (ED; see Methods) explored by each system (Figure 3A) and computing their alignment with the three major transition directions defined from PC1-2 clusters. Consistent with ENM predictions, we observed that simulations from the closed state tend to spontaneously sample toward the direction of the super-open cluster (51% overlap with 5cfb ↔ 6pm0) more than toward the open-like desensitized area (overlap with 5cfb->5vdh, 0.45%), while simulations from 5VDH tend to relax toward the closed state and barely sample along the open-desensitized axis (76% overlap with 5cfb ↔ 5vdh *versus* 20%). By contrast, all mutants and specially V260M enhance the opening transition displaying even better alignment with 5cfb ↔ 6pm0 (55–60% versus 50%) while they are slightly less efficient sampling in the recovery direction toward the closed state (overlaps 5cfb ↔ 5vdh 70%), especially in the case of TM1-2 loop mutations. These trends are also visible upon MD projection onto PC1-2 to build the corresponding free energy landscapes (Figure 3B) and to examine trajectory time evolution (Figures 4A,B). While WT closed simulations sample one major minima around the starting structure and a minor one skewed toward the direction of super-open structures (Figure 3B top), mutants show one larger elongated minimum shifted again toward the same super-open direction. Inspection of the simulation PC1-2 time evolution reveals that indeed a fraction of 5CFB trajectories proceed along PC1(blooming) and PC2 (gating) toward un-blooming/pore opening directions characteristic of the open/desensitized state, especially in the case of V260M (Figure 4A). By contrast, WT desensitized simulations show two small minima in the direction of 5CFB closed state (at 0.0), while mutant ones, also sampling across the same PC1 direction, point toward lower PC2 values, which are only explored in closed mutant simulations (see below). Accordingly, all 5VDH trajectories uniformly proceed along PC1 toward 5cfb minima (Figure 4B), although in terms of PC2 pore gating, the majority evolve toward the super-open cluster. Overall, this suggests a general trend for mutants to favor channel opening while slowing down recovery from desensitization, as is often reported for HPX mutations. Interestingly, although the biological relevance of super-open structures has been questioned, projections onto PC1-2 space clearly show that all simulations from the closed state are sampling PC2 toward solved super-open structures, while simulations from the desensitized 6PMO sample mostly PC1 toward the closed state cluster.

**FIGURE 5:**
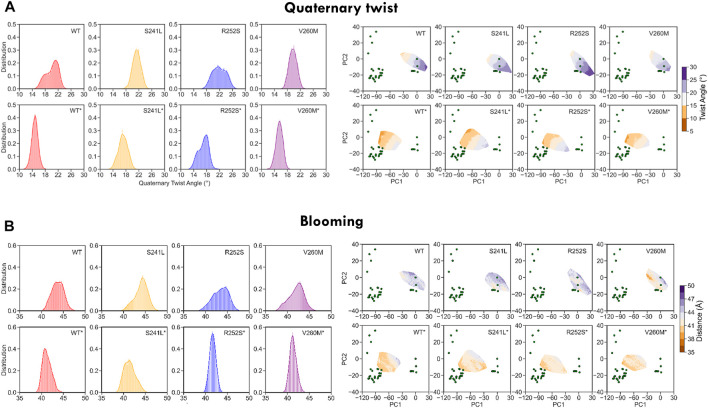
WT and mutant GlyR global ECD dynamics from 1.2 us meta-trajectories. **(A)** Global ECD-TMD quaternary twist angle and **(B)** blooming. Note that the most global descriptor of channel status, quaternary twist, preserves a clear gradient-like pattern across the PC1-2 space both in WT and mutant simulations. See average values in Supplementary Table S3.

**FIGURE 6:**
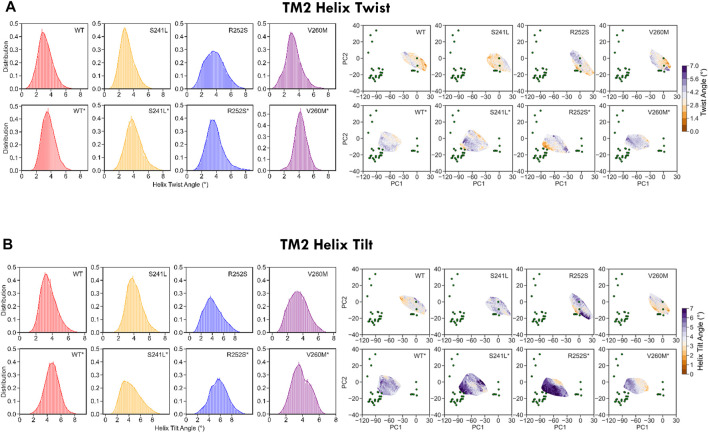
WT and mutant GlyR local TM2 features extracted from 1.2 us meta-trajectories. **(A)** TM2 helix twist and **(B)** TM2 helix tilt. Mutations perturb not only TM2 features but also display long-range allosteric effects on ECD blooming versus WT. See average values in Supplementary Table S3.

Finally, to better characterize the conformational effect of mutations, we also examined the classical heuristic variables describing PLGIC geometry: quaternary twist, blooming, and TM2 tilt and twist angles (Supplementary Table S3 *,* Figures 5, 6). As could be expected, the ECD quaternary twist (Figure 5A) was higher not only for closed versus open state simulations but also for mutants versus WT trajectories, displaying in all cases a neat gradient across PC1. However, less marked gradients were observed for the rest of channel parameters, as could be expected from their poorer correlations with PCs (Supplementary Figure S2B). Apart from the quaternary twist, the ECD configuration is also defined by its maximum radius or blooming, which was shifted to the right by all mutations in both closed and desensitized simulations (Figure 5B). Interestingly, ECD blooming appeared more constrained, i.e., un-bloomed (higher peaks) in TM2 R252S* and V260M*, in comparison with S241L*. The impact of mutations was also apparent in local TM2 variables like helix twist and tilt (Figures 6A,B), especially upon projection, which revealed heterogeneous distributions across the PC1-2 space. Globally speaking, TM2 twist and tilt increased along PC1 toward the open/desensitized state directions. Nevertheless, while R252S increased tilt values dramatically in desensitized state simulations but also in the closed state, reaching in both cases the highest values (10.5 and 9.9 Å, respectively), V260M displayed relatively low tilt angles, especially in 5VDH simulations. Notably, S241L displayed only a mild increase, only noticeable in 5VDH simulations. Overall, although TM1-2 mutations have a similar mild allosteric effect on ECD features, they profoundly and differently disrupt the local configuration of the pore, sampling extreme values for TM2 twist and tilt angles, which can appear uncorrelated to PC1 and associated blooming and quaternary twist.

### Pore Analysis Shows That TM2 Mutations Perturb Pore Gates and Water-Ion Permeation

**FIGURE 7:**
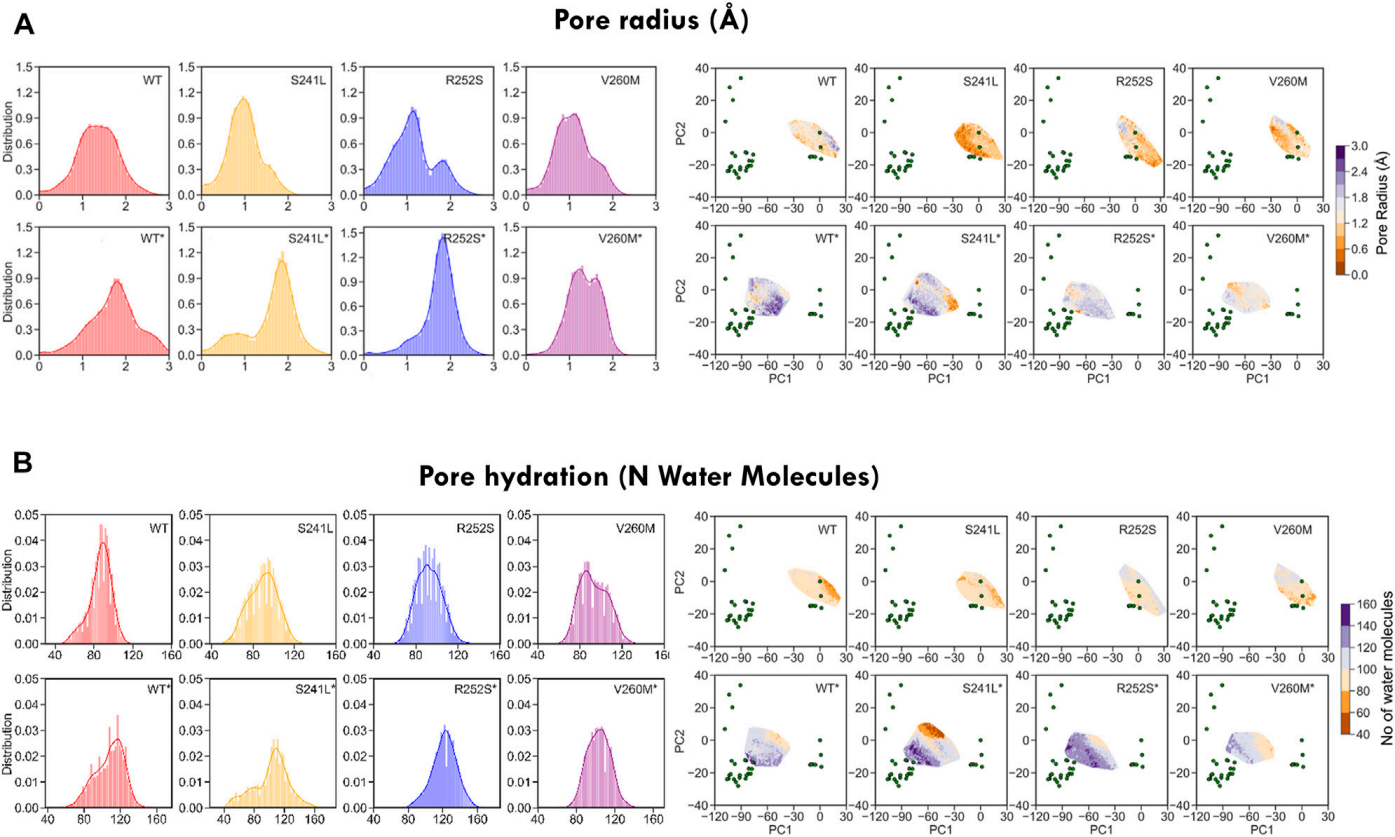
Minimum pore radius **(A)** and hydration **(B)** calculated for WT and mutant GlyR from MD simulations. While WT simulations display Gaussian-like distributions of both pore radius and hydration, mutations shift them toward the right and/or split them in multiple sub-peaks suggestive of enlarged and more hydrated pore states. See average values in Supplementary Table S3.

As these heterogeneous TM2 mutant features suggested a major impact on the pore, we investigated in more detail their effect on pore radius and its permeability for water (Figure 7 *,* Supplementary Table S3). As could be expected from trajectory time evolution (Figure 4B, bottom), WT desensitized simulations from 5VDH, which start with a closed radius of 1.48 Å, quickly evolve opening the pore as seen by broader radius distributions up to a maximum of 3.26 Å (1.7 ± 0.6 Å), in comparison with 5CFB simulations, which start at 1.28 Å and reach up to a maximum of 2.85 Å (1.3 ± 0.5 Å) (see Figure 7A, Supplementary Table S3). Significantly, mutations tend to display broader distributions, generally shifted to the right and with multiple peaks that suggests a diversity of open and closed pore configurations. Differences versus WT simulations are particularly significant for 5CFB simulations, with mutants displaying a clear tail (S241L, V260M) or secondary peak (R252S) at higher nearly open radius close to 2 Å (Figure 7A). This shift to the right is also seen for 5VDH mutant simulations with the exception of V260M; notably, secondary peaks at collapsed pore states barely sampled by the WT are also seen for mutants. These pore radius changes translate to variations in water permeation versus the WT protein. The number of water molecules in the channel ranges from 40 to 120 for the closed state GlyR protein, with an average of ≈80 water molecules in the channel. This seems to shift toward higher values and with a broader distribution for mutant proteins, which have an average of ≈90 water molecules in the channel and maximal values for all mutations around 130–140 (Supplementary Table S3). By contrast, simulations from 5VDH have on average less water molecules in the channel, although in the case of TM2 mutants can reach higher hydration than the WT (up to 170 water molecules for TM1-2 loop mutants versus 160 for WT-GlyR) (Figure 7B).

**FIGURE 8:**
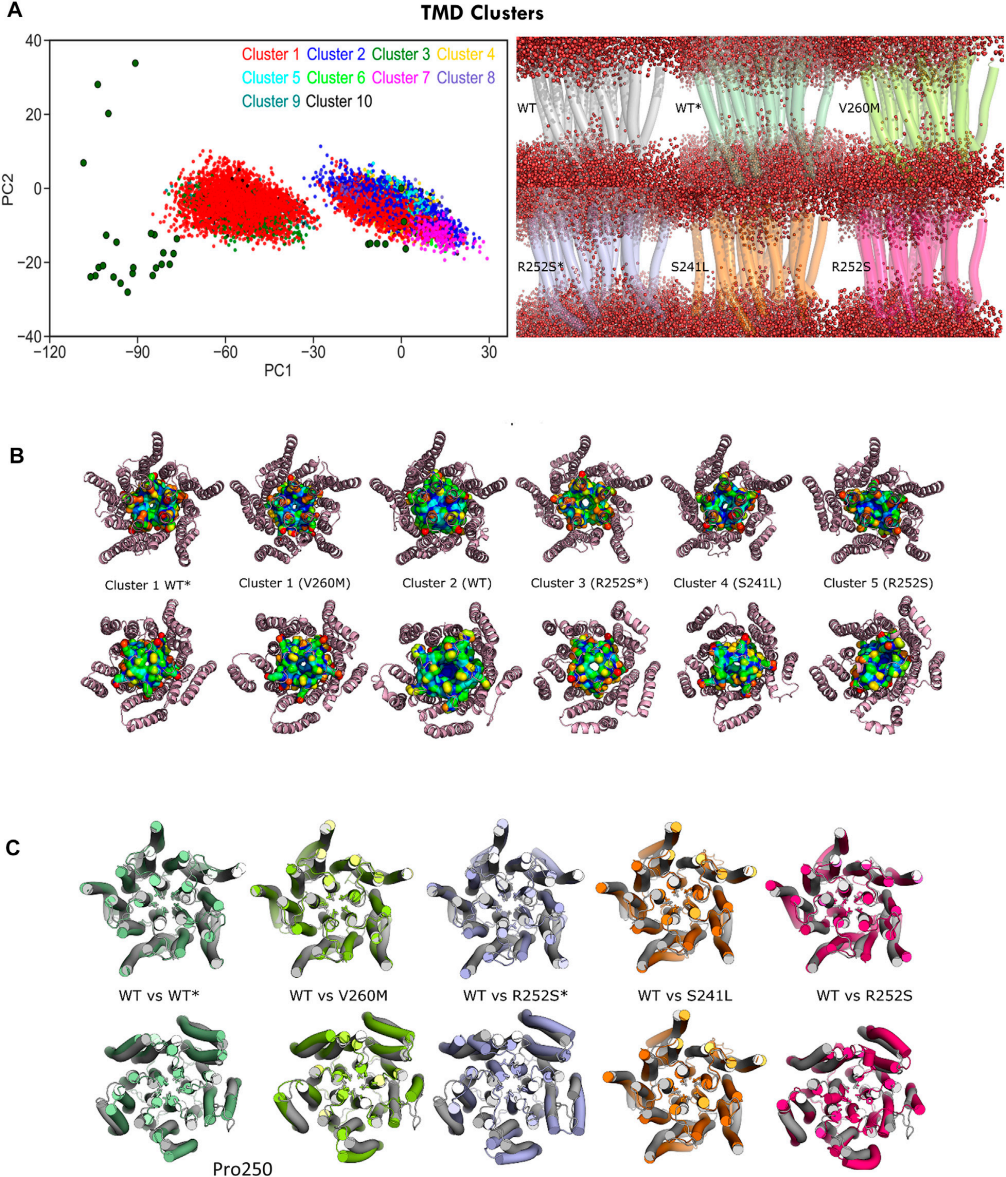
C-alpha RMSD cluster analysis of the GlyR TMD. **(A)** Projections of major clusters onto PC1-2 space (left) and cluster representatives showing water permeation (right): *top row*, cluster 2 (WT) versus cluster 1 (WT* and V260M); *bottom row,* cluster 3 (R252S*), cluster 4 (S241L), and cluster 5 (R252S). **(B)** Top and bottom views of representative TMD clusters and M2 helix residues shown with surface representation. Note the similarity of the open state from 5VDH in WT simulations (WT*) versus the V260M initiated from 5CFB (V260M). **(C)** Top and bottom views of representative TMD clusters, with side chains for pore gates (Leu261, Pro250) shown as licorice. See Supplementary Table S4.

To gain more insight into these differently hydrated pore states explored by MD, we performed TMD cluster analysis on the complete trajectory set with GROMACS (Figure 8, Supplementary Table S4). Simulations initiated from the desensitized state sampled four clusters: cluster 1, which accounts for nearly half of the total conformations sampled by GlyR from closed and desensitized states, and clusters 3, 9, and 10, which in total, account for 7% of the total population and sample expanded pores up to 2Å. Interestingly, we observed that this major semi-open cluster 1 conformation (1.5 ± 0.4 Å) was also sampled by both V260M (13% of cluster 1) and R252S (4% of cluster 1) simulations initiated from the closed state, which allowed the observed increased water permeation (Figure 8A right). Cluster 2 was mostly sampled by WT closed state simulations (51%) as well as the three mutants studied. In contrast, cluster 3, which features an expanded pore around 1.7 Å, is distinctively assigned for R252S* (95% of the cluster). Clusters 5 and 7 are also unique of R252S closed state simulations and feature a slightly collapsed pore (0.8Å), also found in cluster 6, which has a mixed population of conformers from neighbor S241L and R252S mutants (see Supplementary Table S4). Notably, similar collapsed states, previously observed in simulations (Dämgen and Biggin, 2020), are characterized by loss of the fivefold symmetry of the TMD pentamer (cluster 5 in Figures 8B,C). In our simulations, these states accounted, however, for a minor fraction of total sampling and were mostly populated by R252S, which, on the other hand, sampled also expanded pore states like the other mutants as well as WT* trajectories.

**FIGURE 9:**
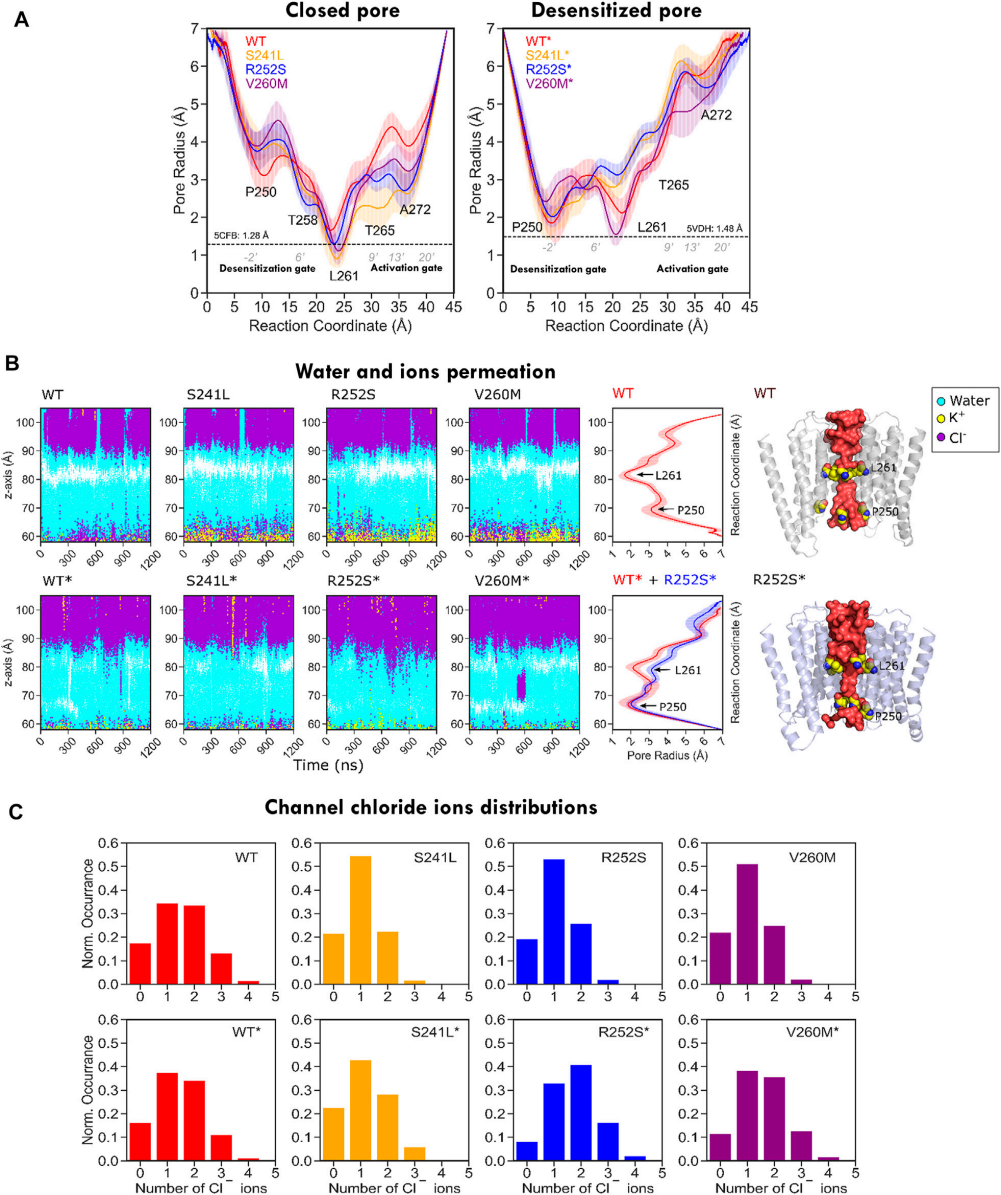
Pore radius profile for WT and mutant GlyR from MD simulations. **(A)** Pore profiles for closed/resting state WT (5CFB) and mutants constructed from it are shown to the left; WT* (5VDH) and mutants constructed from it are shown to the right side. Note how in both closed and desensitized/open state simulations all mutants tend to widen the P250 gate. **(B)** Water and ion permeation across the channel. The closed activation gate in 5CFB simulations (top) appears as a white area around Leu261, missing in desensitized state simulations (bottom). Chloride ions cross the area in WT* and V260M* in relation with permeation events (see Supplementary Movies S1, S2); there is also an entry of potassium ions through the selectivity filter (P250) in closed state simulations (yellow), but they stay in the vestibule. **(C)** Chloride ion distribution inside the channels; note the shift to the right for mutants due to the wider channels accommodating a larger number of ions.

From PLGIC structures and mutagenesis work, it is known that there are two major constriction points in the GlyR pore: an upper ring of hydrophobic residues at the 9′ position of TM2 (the L9′ activation gate, formed by Leu261) and a lower ring of Pro residues at −2′ (the P-2′ desensitization gate, corresponding to Pro250). While the central L9′ gate is closed in antagonist-bound structures (5CFB, 3JAD), in desensitized structures (5VDH, 3JAF) it is P-2′ that closes the intracellular “mouth” of the channel. The P-2′ is wide-open in the so-called super-open structures (3JAE). In general, despite the observed minoritary collapsed TMD clusters, all our simulations resulted in pore relaxation versus the initial crystallographic structures, but with substantial differences upon mutation. A closer inspection of pore profiles (Figure 9A) revealed that TM1-2 mutants dramatically reshape the central channel in a similar way. In the closed state, mutations expand the lower (P250, 9′) but decrease the upper (A272, 20’) pore sections, while in the desensitized simulations, they tend to destabilize and enlarge both. Mutations also displayed wider pore fluctuations, remarkable for S241L and V260M. Although the short time scale of our simulations did not allow us to observe complete opening of the channel, some of the sampled clusters, especially for the mutants, reached pore radius around 1.7–1.8 Å, which can allow the passage of a partially hydrated chloride ion (1.8Å); in such wider pore conformations, water was observed forming continuous molecule chains across the channels. A closer examination of water and ion penetration into the channel revealed that chloride ions penetrate indeed deep into the channel from the enlarged intracellular “mouth”, although only for WT* and V260M* simulations, this resulted in complete permeation events (Figures 9B,C, Supplementary Movies S1, S2). Notably, the enlarged open mouth at the level of the selectivity filter also allowed the entry of potassium ions to the vestibule in 5CFB simulations.

## Summary and Conclusion

Here, we aim to perform a preliminary study of uncharacterized GlyR mutations reported in human tumors. Based on 3D-clustering, conservation, and vicinity or overlap with hyperekplexia changes, we focused on three mutations potentially disrupting gating for further study: one located in the TM1-2 loop, at the “mouth” of the channel (S241L), and two at the back of pore TM2 (R252S and V260M). Our goal was to explore whether they were neutral or perturbed channel dynamics and potentially function. To evaluate functional/conformational status and monitor our simulations, we performed PCA of the current ensemble of GlyR structures, in order to obtain suitable reaction coordinates to build an “experimental” conformational landscape (Orellana et al., 2019a; Orellana, 2019). We confirmed the resulting PCs tracked multiple key channel descriptors providing an automatic and reasonable classification of the solved structures. MD projections on PC1-2 space allowed us to quantify how trajectories sample large-scale motions correlated with multiple channel features. This provided a framework to analyze sampling by MD simulations of human WT and mutant GlyR. Surprisingly, we found evidence that not only the closed-super-open transition is encoded in the experimental structures but also that they are indeed sampled in unbiased WT simulations in the absence of ligand. While WT simulations from the closed state clearly evolve in the direction of super-open solved structures, simulations from the desensitized state tend to sample the recovery transition back to the closed state and not toward the super-open, as suggested by ENM. Our integrated structural analysis also revealed that mutations perturb sampling in a similar way, enhancing the exploration of the opening direction and disrupting relaxation to the native closed state. These global features relate to the local disruption of TM1-2 contacts, which results in an altered TM2 orientation that favors a wider pore and increased water permeation. Nevertheless, the impact of mutations was clearly distinct, with the mutation R252S, which targets the most conserved residue, resulting in non-native interactions and sampling of collapsed pore states. On the contrary, mutation V260M was the closest in behavior to the closed state and displayed enhanced sampling of the opening transition. Importantly, chloride permeation was only observed for WT* and V260M* simulations from the desensitized state. Functional evidence suggests that the mutation of Arg252 results in non-conducting channels, which would agree with our observations of a profoundly impaired channel. On contrast, the V260M HPX mutant has been shown to result in spontaneous activity and prolonged desensitization, also in agreement with our simulations. Functional data for mutations surrounding S241L are still lacking, but our results suggest a behavior in between the other two, probably closer to WT and V260M in terms of channel permeability and conduction despite its proximity to R252S.

In summary, our analysis indicates, on one side, that the concentration of GLRA1-3 mutations across TM1 and TM2 is very likely not random, and, on the other, that these changes certainly disrupt the GlyR function in the tumor cells carrying them, as seen for HPX. Whether and how disrupting GlyR function can represent and advantage for cancer cells is beyond the scope of this work but certainly deserves further computational as well as functional investigation.

## Data Availability

The data and code used for this study are available from the corresponding author LO upon request.
