# Supplementary material for: Exploring the Conformational Impact of Glycine Receptor TM1-2 Mutations Through Coarse-Grained Analysis and Atomistic Simulations
Source: Front Mol Biosci. 2022 Jun 28;9:890851. doi: 10.3389/fmolb.2022.890851 (PMC9275627; doi:10.3389/fmolb.2022.890851)
Supplement: Supplementary file 3 [file DataSheet1.PDF]

Supplementary Material

1 Supplementary Tables

Table S1. Glycine Receptor hyperekplexia (HPX) versus GLRA3 COSMIC mutations (summary). Sequence neighboring positions in italics, HPX functional effect indicated in brackets when known.

GLRA1 Hyperekplexia	GlyR position	Phenotype	GLRA3 Cancer<br>1546 mutations
R 65 L/W	ECD LOOP D	Recessive	R 65 C/H/S,*Q66H*
R 72 C/H	ECD LOOP D	Recessive	**R72C/H/S**
-	ECD	-	S76I/N
-	ECD	-	P78L/S
-	ECD LOOP A	-	D97N/Y
E 103 K	ECD LOOP A	Recessive	**E103K/G**
Y 128 C	ECD	Dominant (activation)	*L127F*
C 138 S	ECD CYS LOOP	Recessive	**C138S**, *P139Q/S*
M 147 V	ECD CYS LOOP	Recessive	*P146S,* **M147I**
D 165 G	ECD LOOP B	Recessive	*T162K, M163I*
W 170 S	ECD LOOP B	Recessive (↓Zn^{2+} potentiation)	-
-	ECD LOOP C	-	R196P/Q
R 218 Q/W	ECD-TM1	Recessive (impaired gating)	*G221R*
Q 226 E	TM1	Recessive (activation)	*L244P, M227I/K*
Y 228 C	TM1	Recessive	**Y228C**
P 230 S	TM1	Dominant (↑desensitization)	**P230S/L**
S 231 R/N	TM1	Recessive (↓surf. expression?)	*L232V*
W 239 C	TM1	Dominant	*S238Y, V240I, S241L/P*
I 244 N	TM1-2 LOOP	Recessive (↑desensitization)	-
P 250 T	TM1-2 LOOP	Dominant (↑desensitization)	*A249T*
R 252 H/C	TM1-2 LOOP	Recessive (↓surf. expression?)	R252S
G 254 D	TM2	Recessive	-
V 260 M	TM2 pore	Dominant (impaired gating)	*T259A,* **V260M**, T262M
T 265 I	TM2 pore	Dominant (impaired gating)	-
Q 266 H	TM2 pore	Dominant (impaired gating)	-
S 267 N	TM2 pore	Dominant (impaired gating)	**S267N**
S 270 T	TM2	Dominant (impaired gating)	**-**
R 271 L/Q/P/X	TM2-3 LOOP	Dominant (impaired gating)	**-**
K 276 E/Q	TM2-3 LOOP	Dominant (impaired gating)	**-**
Y 279 C/S/X	TM2-3 LOOP	Dominant (impaired gating)	*S278L*
V 280 M	TM3	Dominant (activation)	*K281N, A282D/P*
L 291 P	TM3	Dominant	*L292F*
G 342 S	TM3-4 LOOP	Dominant	*D341N*
R 392 H	TM4	Dominant (↓surf. expression?)	*R391G/Q*
R414H	TM4	Dominant (activation)	*F413L*

Table S2. Summary of simulations.

Starting structure	Variant	Number of atoms	Replica - Simulation Length	RMSD AVG +SD
5CFB (closed)	WT	203320	1 300ns<br>2 300ns<br>3 300ns<br>4 300ns	2.33±0.27<br>2.43±0.28<br>2.20±0.26<br>2.35±0.20
	S241L	202649	1 300ns<br>2 300ns<br>3 300ns<br>4 300ns	2.35±0.23<br>2.27±0.23<br>2.44±0.36<br>2.47±0.28
	R252S	202827	1 300ns<br>2 300ns<br>3 300ns<br>4 300ns	2.61±0.24<br>2.51±0.22<br>2.49±0.22<br>2.54±0.30
	V260M	203315	1 300ns<br>2 300ns<br>3 300ns<br>4 300ns	2.33±0.33<br>2.26±0.24<br>2.44±0.30<br>2.14±0.14
5VDH (desensitized)	WT*	192889	1 300ns<br>2 300ns<br>3 300ns<br>4 300ns	2.06±0.19<br>2.30±0.33<br>2.35±0.32<br>2.26±0.36
	S241L*	188226	1 300ns<br>2 300ns<br>3 300ns<br>4 300ns	2.24±0.22<br>2.61±0.41<br>2.35±0.33<br>2.65±0.30
	R252S*	188069	1 300ns<br>2 300ns<br>3 300ns<br>4 300ns	2.38±0.27<br>2.67±0.28<br>2.60±0.25<br>2.62±0.32
	V260M*	188161	1 300ns<br>2 300ns<br>3 300ns<br>4 300ns	2.38±0.38<br>2.16±0.26<br>2.24±0.19<br>2.27±0.26

Table S3. Avg ± SD for receptor heuristic variables in MD 1200 ns metatrajectories

	RMSD (Å)	*QTwist (Å)*	*Bloom (Å)*	*TM2 Twist(°)*	*TM2 Tilt(°)*	*Pore radius(Å)*	*Pore water*
Closed							
WT	2,3 ± 0,3	20,6 ± 2,0	43,6 ± 1,3	3,2 ± 1,0	3,6 ± 1,0	1,3 ± 0,5	86 ± 12
S241L	2,4 ± 0,3	21,0 ± 1,4	44,0 ± 1,5	3,0 ± 0,9	4,0 ± 1,0	1,0 ± 0,4	89 ± 14
R252S	2,5 ±0,3	22,0 ± 1,9	43,3 ± 1,7	3,7 ± 1,2	4,2 ± 1,5	1,1 ± 0,5	93 ± 12
V260M	2,3 ± 0,3	19,7 ± 1,2	42,0 ± 1,7	3,2 ± 1,0	3,4 ± 1,2	1,1 ± 0,4	93 ± 14
Desensitized							
*WT**	2,8 ± 0,15	15,1 ± 1,1	41,2 ± 1,0	3,6 ± 0,8	4,7 ± 1,0	1,7 ± 0,6	108 ± 16
*S241L**	2,7 ± 0,2	16,5 ± 1,7	41,4 ± 1,3	4,0 ± 1,0	4,4 ± 1,6	1,6 ± 0,6	103 ± 23
*R252S**	2,8 ± 0,15	17,1 ± 1,5	41,6 ± 0,7	3,8 ± 1,2	5,7 ± 1,5	1,7 ± 0,4	122 ± 14
*V260M**	2,7 ± 0,2	15,4 ± 1,0	41,3 ± 0,8	4,2 ± 0,8	3,8 ± 1,0	1,4 ± 0,4	103 ± 12

Table S4. Representative RMSD clusters from total meta-trajectory containing WT and mutant simulations. Only the TM domain C-alpha backbone is considered for rMSD calculation.

N	Frames	% Total Population	Variants		PC1-2 center	Avg pore (Å)	Avg blooming (Å)	Quaternary twist Angle (º)
1	4997	51% Semi-open Un-bloomed	4% R252S 13% V260M	22% WT* 21% S241L* 11% R252S* 24%V260M*	-45.6, -4.6	**1.5±0.4**	41.6±2.0	16.7±1.3
2	2168	22% Closed	51% WT 22% S241L 6% R252S 19% V260M	-	-2.4, -4.0	1.2±0.3	43.5±2.1	20.5±1.4
3	629	6% Semi-open	-	3% WT* 95% R252S*	-52.1, -7.4	**1.7±0.4**	41.5±2.0	17.1±1.3
4	567	5% Closed	81% S241L 18% R252S	-	-0.6, -5.0	1.2±0.3	44.3±2.1	20.8±1.4
5	294	3% Closed	100% R252S	-	-0.8, -3.7	0.8±0.3	41.4±2.0	21.4±1.5
6	227	2% Semi-open Bloomed	27% S241L 72% R252S	-	0.9, -7.2	**1.6±0.4**	43.6±2.1	21.1±1.5
7	225	2% Fully Closed Bloomed	100% R252S	-	11.4, -12.3	0.8±0.3	43.6±2.1	24.3±1.6
8	188	1% Fully Closed Bloomed	15% WT 32% S241L 52% V260M	-	-5.7,-3.4	1.0±0.3	42.7±2.1	21.0±1.5
9	101	1% Semi-open	-	12% WT* 59% S241L* 24% R252S* 2% V260M*	-52.5, -4.1	1.4±0.4	40.8±2.0	16.4±1.3
10	74	0.01% Semi-open Un-bloomed	-	22% WT* 36% S241L* 40% R252S*	-55.2, -4.7	**1.8±0.4**	41.9±2.1	16.3±1.3

Supplementary Figures

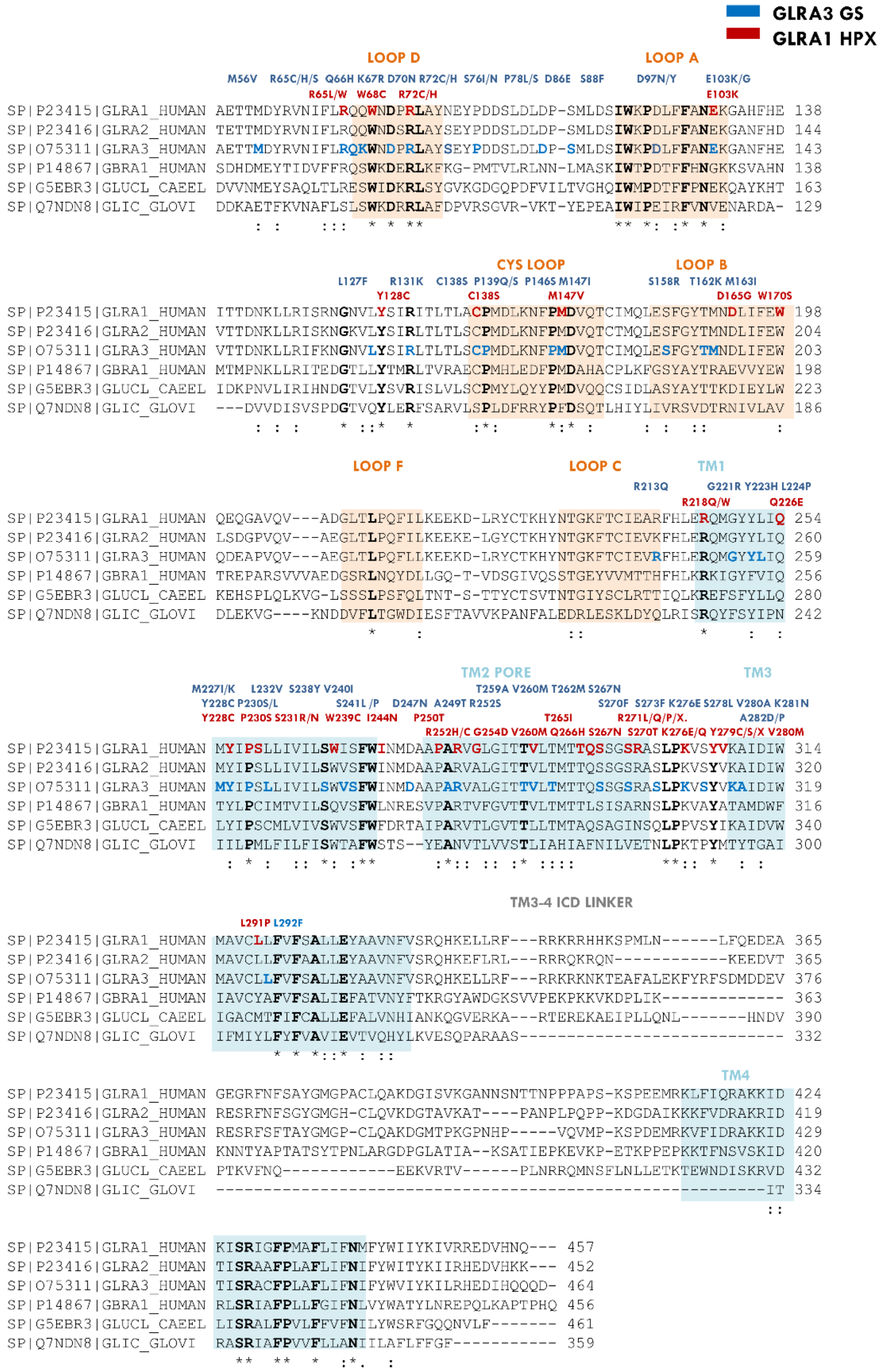

Supplementary Figure 1. ClustalW alignment of GLRA1-3, GBRA1, GluCL and GLIC sequences highlighting GLRA1 hyperekplexia and GLRA3 COSMIC mutations.

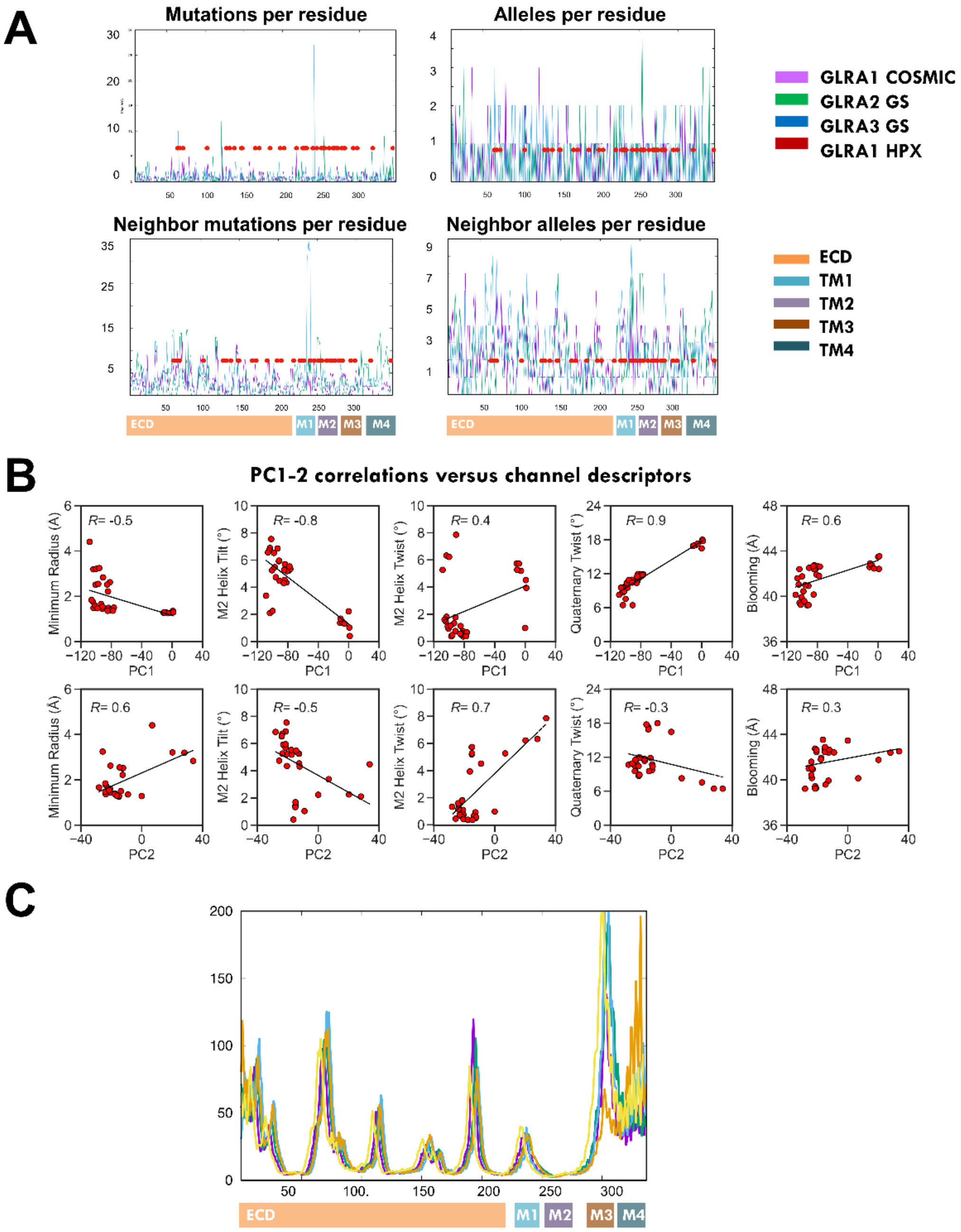

Supplementary Figure 2. GlyR raw mutational frequency, PCA & ENM. A) *Upper row:* raw number of mutations reported per position (*left*) and number of allelic variants reported per position (*right*); *Lower row:* number of neighbor mutations (*left*) and number of neighbor allelic variants (*right)* within a sphere of 10Å of centered on each residue position (see *Methods*). B) Pearson correlations between PC1 and PC2 and channel variables for the experimental GlyR structural ensemble. C) Residue thermal fluctuations computed from the first ten ENM modes of GlyR closed structure 5CFB.

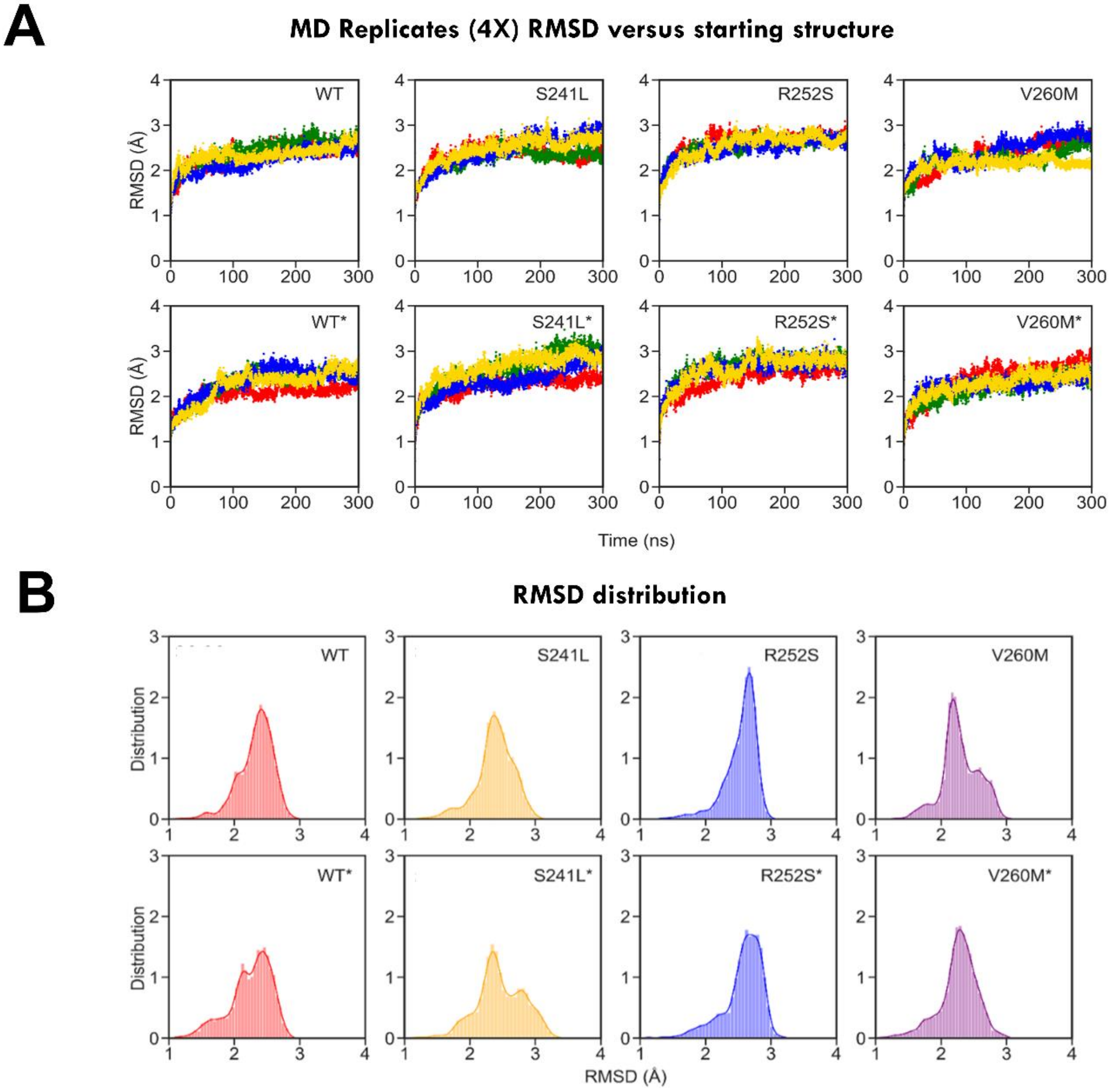

Supplementary Figure 3. RMSD of MD trajectories. A) RMSD time evolution of MD simulation frames upon alignment onto the corresponding starting structure (5CFB for the upper row and 5VDH for the lower), for the 4 replicates run for each condition. B) RMSD frequency histograms for MD simulation frames upon alignment onto starting structures, concatenating all replicates for each condition (1,2 μs trajectories). Mutations shift RMSD distributions to the right revealing additional peaks suggestive of wider conformational sampling and flexibility.

A

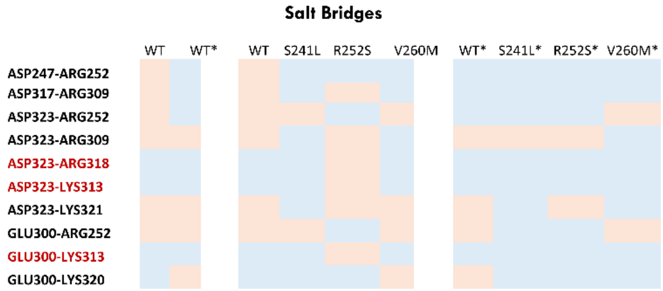

B

Hydrogen bonds (only mutated residues)

Donor	Acceptor	WT	WT*	WT	S241L	R252S	V260M	WT*	S241L*	R252S*	V260M*
SER241-Main	LEU237-Main	70.2	0.0	70.2	78.1	77.7	57.1	0.0	80.8	0.0	55.0
SER241-Side	LEU237-Main	77.2	0.0	77.2	0.0	55.3	81.5	0.0	0.0	0.0	0.0
SER241-Side	SER238-Main	80.3	77.4	80.3	0.0	73.3	87.7	77.4	0.0	61.1	78.7
SER241-Side	ARG252-Main	0.0	54.5	0.0	0.0	0.0	0.0	54.5	0.0	58.6	82.8
SER241-Side	SER238-Main	0.0	72.3	0.0	0.0	0.0	0.0	72.3	0.0	92.5	74.2
ILE244-Main	SER241-Main	90.2	94.5	90.2	94.1	68.4	95.4	94.5	95.1	91.6	97.5
ARG252-Main	ALA248-Main	95.8	98.1	95.8	99.7	87.4	97.0	98.1	99.7	96.6	99.5
ARG252-Side	ASP323-Side	53.8	99.8	53.8	83.8	0.0	0.0	99.8	87.8	0.0	100.0
ARG252-Side	GLU300-Side	99.7	0.0	99.7	98.3	0.0	99.4	0.0	96.5	0.0	0.0
ARG252-Side	ILE244-Main	84.7	82.5	84.7	90.8	0.0	79.8	82.5	0.0	0.0	91.2
GLY256-Main	ARG252-Main	98.6	91.8	98.6	99.4	97.8	99.2	91.8	99.4	99.6	88.6
VAL260-Main	GLY256-Main	99.9	99.8	99.9	99.2	99.9	100.0	99.8	99.9	99.5	100.0
THR264-Main	VAL260-Main	99.8	99.9	99.8	99.9	99.9	100.0	99.9	99.9	99.9	100.0
THR264-Side	VAL260-Main	99.9	99.8	99.9	100.0	99.8	99.7	99.8	99.8	98.4	99.8

C

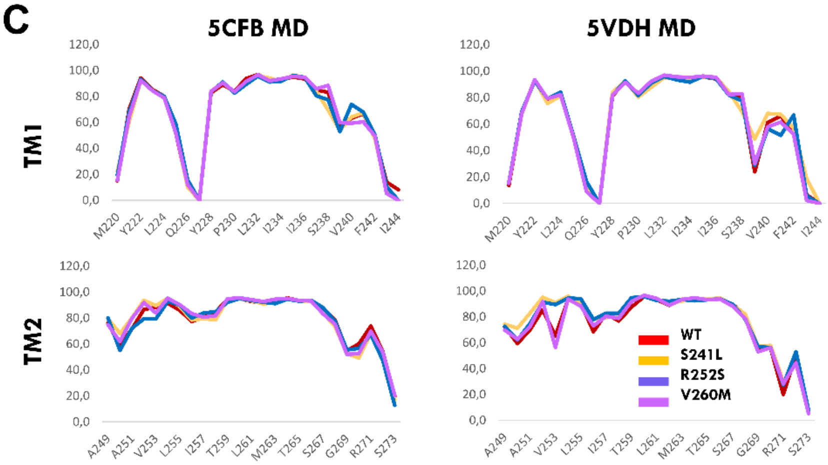

6 **Supplementary Figure 4. Salt bridges and hydrogen bonds & TM1-2 Helicity.** A) Salt bridges characteristic of closed (WT) and desensitized state (WT*) simulations (rows 1-2) versus mutants in closed state (rows 3-6) and desensitized state simulations (rows 7-10). Non-native bonds in WT simulations formed in R252S are highlighted in red. B) Hydrogen bonds involving mutated residues. Bonds present throughout most of the simulation time in pink, absent in blue, occupancy indicated for H-bonds. C) TM1 and TM2 average helicity in simulations from the closed (WT, S241L, R2525S, V260M) and desensitized state (WT*, S241L*, R252S*, V260M*)
